# Development of Salmon Sperm DNA/Regenerated Silk Bio-Based Films for Biomedical Studies on Human Keratinocyte HaCaT Cells under Solar Spectrum

**DOI:** 10.3390/jfb14050280

**Published:** 2023-05-18

**Authors:** Maria Rachele Ceccarini, Francesca Ripanti, Veronica Raggi, Alessandro Paciaroni, Caterina Petrillo, Lucia Comez, Kevin Donato, Matteo Bertelli, Tommaso Beccari, Luca Valentini

**Affiliations:** 1 Department of Pharmaceutical Sciences, University of Perugia, 06123 Perugia, Italy; 2 Department of Physics and Geology, University of Perugia, Via Alessandro Pascoli, 06123 Perugia, Italy; 3 Polo Scientifico Didattico, University of Perugia, Strada di Pentima 4, 05100 Terni, Italy; 4 Istituto Officina dei Materiali-IOM, National Research Council-CNR, Via Alessandro Pascoli, 06123 Perugia, Italy; 5 MAGI EUREGIO SCS, Via Maso della Pieve, 60/A, 39100 Bolzano, Italy; 6 MAGISNAT, Atlanta Tech Park, 107 Technology Parkway, Peachtree Corners, GA 30092, USA; 7 Civil and Environmental Engineering Department, University of Perugia, Strada di Pentima 6, 05100 Terni, Italy

**Keywords:** regenerated silk, DNA, biopolymers, biomedical devices, cell metabolism

## Abstract

In this study, we fabricated adhesive patches from silkworm-regenerated silk and DNA to safeguard human skin from the sun’s rays. The patches are realized by exploiting the dissolution of silk fibers (e.g., silk fibroin (SF)) and salmon sperm DNA in formic acid and CaCl_2_ solutions. Infrared spectroscopy is used to investigate the conformational transition of SF when combined with DNA; the results indicated that the addition of DNA provides an increase in the SF crystallinity. UV–Visible absorption and circular dichroism spectroscopy showed strong absorption in the UV region and the presence of B-form of DNA once dispersed in the SF matrix, respectively. Water absorption measurements as well as thermal dependence of water sorption and thermal analysis, suggested the stability of the fabricated patches. Biological results on cellular viability (MTT assay) of keratinocyte HaCaT cells after exposures to the solar spectrum showed that both SF and SF/DNA patches are photo-protective by increasing the cellular viability of keratinocytes after UV component exposure. Overall, these SF/DNA patches promise applications in wound dressing for practical biomedical purposes.

## 1. Introduction

Recyclable and biodegradable materials consisting of biopolymers, such as starch, casein, and lignin, have been recently reported [1,2,3,4,5,6] as a possible solution to reduce plastic pollution. An intriguing possibility could be to exploit the combination of different bio-materials for specific applicative uses [7].

Deoxyribonucleic acid (DNA), a natural polymer that can be extracted from living organisms, has unique physiological and optical properties [8,9], and it is available in large amounts considering that approximately 50 billion metric tons are actually discarded [10]. It is well known that ultraviolet light can damage DNA within the skin [11] but, exploiting this weakness, it can be engineered as a sacrificial layer by flipping the approach and using DNA as a layer to protect the epidermis cells. In this regard, salmon milt DNA-based bioplastic has been recently reported [12]. Since DNA is water-soluble and has UV absorption ability, recycling this industrial waste can be considered an opportunity to produce functional biomaterials in a sustainable way.

Silk fibroin (SF) is another inexhaustible biopolymer that can be extracted from silkworms [13]. The regenerated silk can be produced with different content of crystalline domains with β-sheet conformation, and, as a result, a biopolymer can be obtained with water insolubility, thermal stability, and UV absorbing properties [14,15]. 

UV light radiation (e.g., 230–400 nm) accounts for approximately 6.8% of the solar spectrum radiation reaching the Earth’s surface [16]. Solar UV radiation contributes to a variety of skin damages since it penetrates the skin in a wavelength-dependent manner [17] with UVA (320–400 nm) that thoroughly enters the dermis, UVB (280–320 nm) that is almost completely absorbed by the epidermis and, finally, UVC (230–280 nm) that is the less absorbed component [18,19,20]. Solar UV radiation is the most important risk factor for skin disorders [21], including inflammation, degenerative aging, and cancer (basal cell carcinoma, squamous cell carcinoma, and malignant melanoma are the three most common types) [22]. On the other hand, vitamin D (cholecalciferol) natural production by the chemical conversion of 7-dehydrocholesterol in the skin is possible only after UVB exposure [23,24]. A current challenge is to maintain adequate vitamin D production while minimizing the risk of skin cancer, even when using sunscreen that blocks UVB absorption [25]. In fact, vitamin D is involved in a plethora of biochemical pathways essential for human health: from wound healing [26] to infections such as SARS-CoV-2 [27].

Actually, cosmetics and sunscreens are produced by adding metal-oxide nanoparticles to shield against UV radiation. Demokritou et al. [28], for example, found that zinc oxide nanoparticles often added to creams to block UV rays can damage DNA. The bottleneck of such nanoparticles relies on the production of reactive oxygen species, which produce oxidative stress, overwhelming innate antioxidant benefits. Oxidative stress is responsible for a wide range of damage to DNA, including cytotoxic or mutagenic effects [28,29]. Hence, by combining DNA salmon sperm with the other natural photoprotective biopolymer (SF), we developed photoprotective bio-based films. In particular, we realized adhesive sunscreens prevent the damage produced by UV radiation on epidermis cells. The facile processing method proposed might inspire the development of alternative and more sustainable materials reducing the reapplication time of actual sunscreen chemicals on the skin and thus their dispersion on the environment [30].

## 2. Materials and Methods

### 2.1. Materials

Silk cocoons were supplied by a local farm (Fimo srl, Milano, Italy). Sodium hydrogen carbonate (NaHCO_3_), CaCl_2_, and formic acid (FA) were provided by Merck (Darmstadt, Germany). Salmon sperm DNA (double-stranded DNA, molecular weight = 5 × 10^3^ g/mol, 23 base pairs) was purchased from Merck (Darmstadt, Germany). Human keratinocytes (HaCaT cell lines) were purchased from I.Z.S.L.E.R. from the Istituto Zooprofilattico Sperimentale della Lombardia e dell’Emilia Romagna ‘Bruno Ubertini’ (Brescia, Italy). Cells were grown in 75 cm^2^ tissue flasks with DMEM medium supplemented with 100 U/mL penicillin, 100 μg/mL streptomycin, 2 mM L-glutamine, and 10% FBS under a humidified atmosphere of 5% CO_2_ at 37 °C.

### 2.2. Synthesis of SF/DNA Films

Regenerated silk was produced according to a protocol described elsewhere [31]. Briefly, 1.42 g of degummed *Bombyx mori* silk fibers were dissolved in 25 mL of FA containing 0.95 g of CaCl_2_ in a 60:40 ratio at 30 °C for 1 h. Then, 0.4 mL of DNA water solutions with different concentrations (e.g., 1.3 mg/mL, 2 mg/mL, 2.6 mg/mL, 3.3 mg/mL, and 4 mg/mL, respectively) were added into the SF/FA solution and mechanically stirred for 30 min. Films were finally obtained by drop-casting the solutions into Petri dishes with a diameter of 5 cm and left to evaporate at 40 °C for 2 h. From now on, SF/A, SF/B, SF/C, SF/D, and SF/E refer to SF/DNA samples prepared by adding the five concentrations of DNA (e.g., from 1.3 mg/mL to 4 mg/mL) to the SF/FA solution. 

### 2.3. Materials Characterization

To perform UV–Visible (UV–Vis) absorption and circular dichroism (CD) measurements, film samples were dropcasted on a quartz window of 1 mm thickness after redispersing them in FA. UV–Vis absorption experiments were performed using a Jasco V-570 spectrophotometer, exploring the spectral range from 200 nm to 350 nm, where the characteristic absorptions of both silk and DNA are present. CD experiments were carried out with a Jasco J-810 spectropolarimeter on the same films on the quartz window in the 200–350 nm spectral range, with a 50 nm/min scan speed. 

Attenuated total reflection (ATR) Fourier transform infrared (FTIR) measurements were performed using a Jasco spectrometer. The spectral range between 400 and 4000 cm^−1^ was scanned with a resolution of 2 cm^−1^. In the 1450–1750 cm^−1^ spectral range, amide I and amide III crystallinity index (CI) values were determined by calculating the ratios of the absorbance at 1620 cm^−1^/1652 cm^−1^ for amide I and 1264 cm^−1^/1230 cm^−1^ for amide III, respectively. Fourier deconvolution of the ATR-FTIR was performed by Origin 9.0 software. Lorenzian line shape was used for the deconvolution of the spectra; the positions of the bands were kept fixed when using the autofit program of the software. Then a straight baseline was subtracted. Assuming the same extinction coefficient for the different stretching modes, the spectra were then curve-fitted. The band intensities are proportional to the fraction of amide I and amide III structural components.

The swelling of the SF and SF/DNA samples was calculated as follows:

(W_s_ − W_0_)/W_0_ × 100 (1)

where W_0_ and W_s_ are the initial and soaked weights in phosphate-buffered saline (PBS, pH 7.5 at 10 °C) of the sample, respectively.

The film stability was measured in two different conditions: at a fixed relative humidity (RH), varying the temperature from 25 °C to 75 °C, and at a fixed temperature (e.g., 75 °C), varying RH from 20% to 80%. The water uptake was calculated as the ratio between the mass before and after water vapor sorption.

The current–voltage (I–V) curves were recorded by a Keithley 4200 SCS on SF and SF/DNA rectangular samples fixed between two stripes of adhesive copper. 

The shear strength was determined by a lap shear test that allows the calculation of the bonding of materials when tested on a joint specimen. The test requires the bonding of two rigid plastic with the adhesive. The two specimens are overlapped to detect the failure in the adhesive.

In our experiments, two rigid films of poly(3-hydroxybutyrateco-3-hydroxyvalerate) were used and adhered by applying a force on SF and SF/DNA films (e.g., 1N). The shear strength was determined by dividing the ultimate tensile strength, calculated by the tensile machine, by the adhesion area. The overlapped area was 10 × 10 mm^2^, and the load was applied with a strain rate of 5 mm/min (the results were the average values from at least three measurements for each composition).

### 2.4. Irradiation and MTT Assay 

Cell viability was evaluated by MTT assay [32]. HaCaT cell line, used as a representative model of the epidermis, was grown in DMEM (4.5 g/L glucose, 584 mg/L L-glutamine, 3.7 NaHCO_3_), with 10% FBS and 0.1% Pen/Strep at 37 °C in the presence of 5% CO_2_. Cells were seeded in polystyrene tissue culture dishes of 35 × 10 mm diameter. The final concentrations depend on times: specifically, 4 × 10^5^ cells were seeded for cells harvested 4 h post-irradiation, 3 × 10^5^ for cells grown for 24 h post-irradiation, 2 × 10^5^ for 48 h post-irradiation, and finally 1 × 10^5^ for 72 h post-irradiation. Cells were irradiated in PBS 1X pre-warmed in the air at room temperature with a 300 W Xenon light (ThermoOriel solar simulator model 69907). The average power in a 33 mm diameter output beam in the wavelength range of UVC, UVB, UVA, and VIS-nIR (400–780 nm) was 11.5 mW, 27 mW, 85 mW, and 430 mW, respectively. The doses of radiation during 220 s exposure were 2.96 kJ/m^2^ (UVC), 6.95 kJ/m^2^ (UVB), 21.9 kJ/m^2^ (UVA), and 110 kJ/m^2^ (VIS-nIR), respectively. Negative control was kept in PBS 1X for the same times as irradiated samples. Positive control was kept under the solar spectrum without a protective device. All other samples were covered by SF or SF/DNA in different concentrations. Immediately after irradiation, PBS 1X was removed and changed with 1 mL of complete culture medium. After 4, 24, 48, and 72 h, 100 µL of MTT (5 mg/mL stock) was added to each dish (0.5 mg/mL final concentration) and, after 3 h of incubation at 37 °C with 5% CO_2_, the supernatant was carefully removed, and 1 mL DMSO was added to lysed cells to allow the formazan complete dissolution. After 30 min, using an automatic microplate reader (Eliza MAT 2000, DGR Instruments, GmbH), the absorbance values (OD) were measured spectrophotometrically at λ_max_ = 540 nm in triplicates per well and quadruplicates (biological replicates) per condition. Cell viability, normalized to the corresponding negative control, was calculated as previously described [32,33]. 

### 2.5. Statistical Analysis

GraphPad Prism 9.2.0.332 (GraphPad Software, San Diego, CA, USA) was used to assess statistical significance. A two-way ANOVA with Tukey’s post-hoc analysis was performed. 

## 3. Results and Discussion

**Figure 1:**
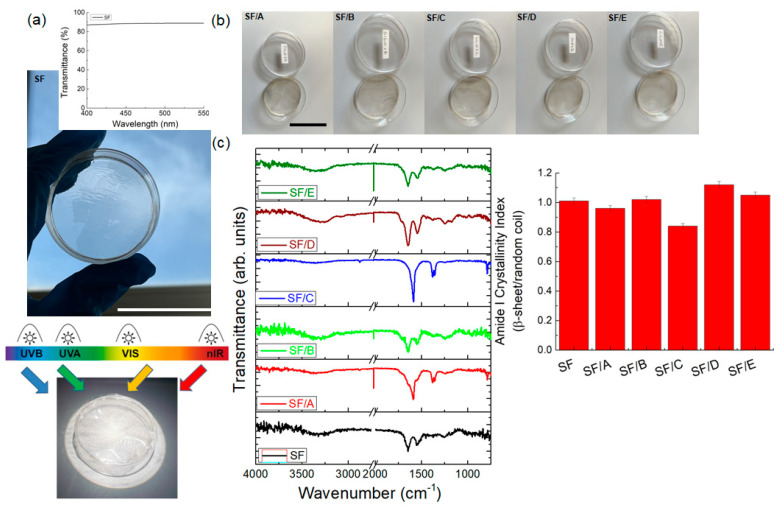
(**a**) Photograph with transmittance spectrum of the SF film in the visible wavelength range and highlight of the proposed application depicting the placement of the cells covered by the SF/DNA patch as filter for the individual beams. (**b**) Photographs of the SF/DNA prepared samples. The scale bars indicate 5 cm. (**c**) ATR-FTIR spectra and amide I crystallinity index of SF and SF/DNA samples. Triplicate experiments gave similar results.

The SF film showed an optical transmittance of 88% in the visible range, as well as SF/DNA samples, providing an optical transparency that is enough for a view of the human skin (Figure 1a,b). We first investigated the changes in IR absorption bands of SF and SF/DNA samples, as IR spectroscopy is useful for getting information on the conformational changes of proteins. In particular, the ratios of the intensity of the two amide bands that indicate random/α-helix and β-sheet conformations were used to estimate the crystallinity and the molecular weight of SF (Figure 1c). Therefore, the crystallinity index (CI) was calculated by the ratio of 1620 cm^−1^ (β-sheet) to 1652 cm^−1^ (random coil) for the amide I region [14,34]. According to previous results [35], the SF sample with a CI of 1.01, prepared by dissolving the degummed silk for a relatively short time (30 min), indicated high molecular weight values (>100 kDa). The calculated CI index values for the amide I region (Figure 1c) also indicate that adding DNA to the SF/B, SF/D, and SF/E samples provides a slight increase in the SF crystallinity.

The SF with the DNA addition (e.g., SF/A and SF/C samples) showed absorption bands at 1603 cm^−1^, 1529 cm^−1^, and 1690 cm^−1^ attributed to the scissoring vibration of –NH_2_ in cytosine, guanine, and adenine, respectively [36,37]. The variation of the transmittance obtained by subtracting the signal of the SF film from the spectrum of each SF/DNA film is reported in Figure S1. These absorption bands are attenuated with the DNA concentration increase, suggesting the formation of C-N bonding between the amino groups of the DNA and SF. Finally, due to the hygroscopic behavior of the prepared samples (see below), the broad band observed at around 3220 cm^−1^ is associated with water absorption [38]. The protein backbone groups (e.g., C=O and N-H) can form hydrogen bonds with water at the interface, replacing some of the hydrogen bonds responsible for crystalline domains of SF [38]. 

The effect of DNA concentration on the surface morphology was then analyzed by optical microscopy. The investigation of the SF surface showed smooth characteristics with no specific features, while for the SF/D film, many large structures diffracting the light were observed on the film surface (Figure S2).

**Figure 2:**
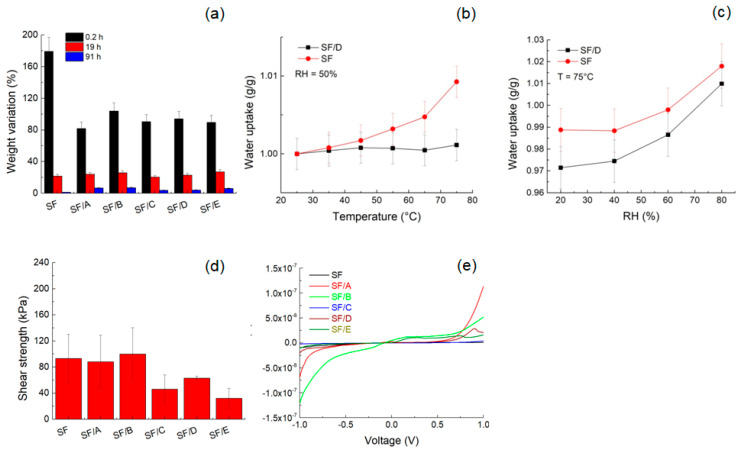
(**a**) Swelling ratio of SF and SF/DNA samples with different DNA content in PBS. The swelling ratio was expressed as an average of five measurements. (**b**) Water uptake of SF and SF/D samples as a function of temperature at RH = 50%. (**c**) Water uptake of SF and SF/D samples as a function of RH at T = 75 °C. (**d**) Shear strength values of SF and SF/DNA samples. (**e**) Current-voltage curves of SF and SF/DNA samples.

The interaction mechanism between the DNA chains and SF was monitored by swelling properties and thermal stability. Figure 2a displays the swelling ratio of SF and SF/DNA samples. The PBS uptake of the SF sample is higher than that of the SF/DNA samples after 12 min. This finding and the recovery of the initial weight indicate both that SF/DNA samples are not soluble in water and that the addition of DNA to SF reduces the hydration process. We further investigated the stability of SF and SF/D samples. With a higher value of the CI, the water uptake of SF/D samples versus temperature does not exhibit a significant variation (Figure 2b). The water uptake isotherms of SF and SF/D samples shown in Figure 2c indicate that, within the statistical error, the RH variation does not lead to an increase of the weight even if recording lower values for the sample with higher CI (e.g., SF/D sample). This result can be rationalized in terms of distinct water sorption, which is more pronounced within a polymer with the prevalence of amorphous chains (e.g., SF), while it is reduced with the transition to a crystalline structure when DNA is added [39]. These results showed how the hybrid SF films follow distinct thermodynamics of water sorption from that of conventional hydrogels. The key parameter is the CI of SF/D film; at a higher temperature, there is a decreased amount of escaped water molecules because of the reduced kinetic energy of crystalline chains. 

Moreover, the SF/A sample after 90 h from being soaked in PBS showed flexibility (Supplementary Video S1). This aspect is of crucial importance when a patch is interfaced with a soft underlying substrate. A qualitative investigation of the adhesiveness of the SF/DNA patches also shows good compliance and adhesion of the SF/A patch to the skin (Supplementary Video S2). These effects are attributed to the presence of the Ca^2+^ ions that form cross-links between random coil chains of silk, strengthening the material, and are water-capturing points [40]. The shear strength of the SF patch (Figure 2d) corresponds to ca. 80 ± 30 kPa, and it decreases with increasing the DNA content. 

The charge transport of the DNA used in these experiments was explained in terms of stacking of nearly parallel bases with overlapping π-electrons [41]. Typical current-voltage curves of both SF and SF/DNA samples are reported in Figure 2e. In general, for low voltages, the ohmic behavior is due to the Boltzmann distribution of the charge carriers and the constant position of the Fermi level [42]. At higher voltages, the non-linear behavior of the current results from the charge-carrier injection [43]. Increasing the DNA content, the overlap of π-electrons is reduced by the loss of the DNA stacking interactions with an overall decrease in electrical conductivity. 

**Figure 3:**
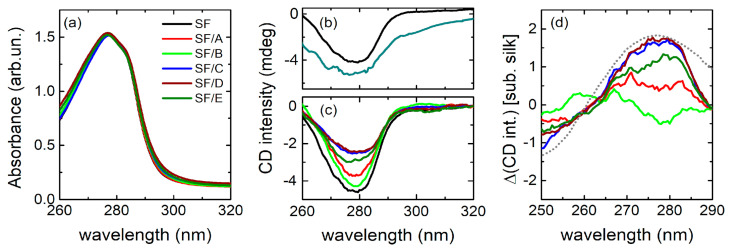
(**a**) UV–Vis spectra of SF and SF/DNA plastics with different concentrations of DNA; (**b**) CD spectrum of SF (black) compared to that of IgG2 protein in a denaturation buffer [43] (cyan); (**c**) CD spectra of SF/DNA samples normalized by the absorption intensity and (**d**) after the subtraction of the CD spectrum of SF; dotted spectrum in this panel represents the DNA in B-form taking from ref. [44].

UV–Vis absorption spectra of SF and SF/DNA films are reported in Figure 3a. The main peak around 280 nm is probably due to the presence of aromatic amino acids distributed along the SF chain, such as tyrosine, phenylalanine, and tryptophan [44]. 

The CD spectrum of SF, reported in Figure 3b, shows a prominent minimum just in correspondence with the peak observed by UV–Vis absorption. In the literature, a similar CD profile is indeed associated with a denaturated form of proteins. As an example, the spectrum of denaturated IgG2 protein is almost perfectly superimposable to that of fibroin in the 260–320 nm range (Figure 3b) [45].

The spectra of SF/DNA samples are shown in Figure 3c. Quite interestingly, the depth of the minimum is strongly dependent on the DNA concentration, indicating that, despite the small fraction in weight, the DNA component provides a significant contribution to the ellipticity. To single out such a contribution, we subtracted the signal of the SF film from the spectrum of each SF/DNA film, as reported in Figure 3d. It turns out that the difference profiles show a maximum at about 275 nm, whose intensity increases with the DNA concentration and whose shape is closely reminiscent of the signal from B-form DNA [46]. This result suggests that the DNA embedded in SF/DNA samples retains a conformation nearly equivalent to the physiological one. Such spectra are similar to those of DNA complexes with divalent ions [47] (Ca^2+^), which interact with phosphates, shielding their charges and altering the hydration properties of DNA. However, Figure 3d shows that the occurrence of these interactions does not affect the DNA CD spectrum.

**Figure 4:**
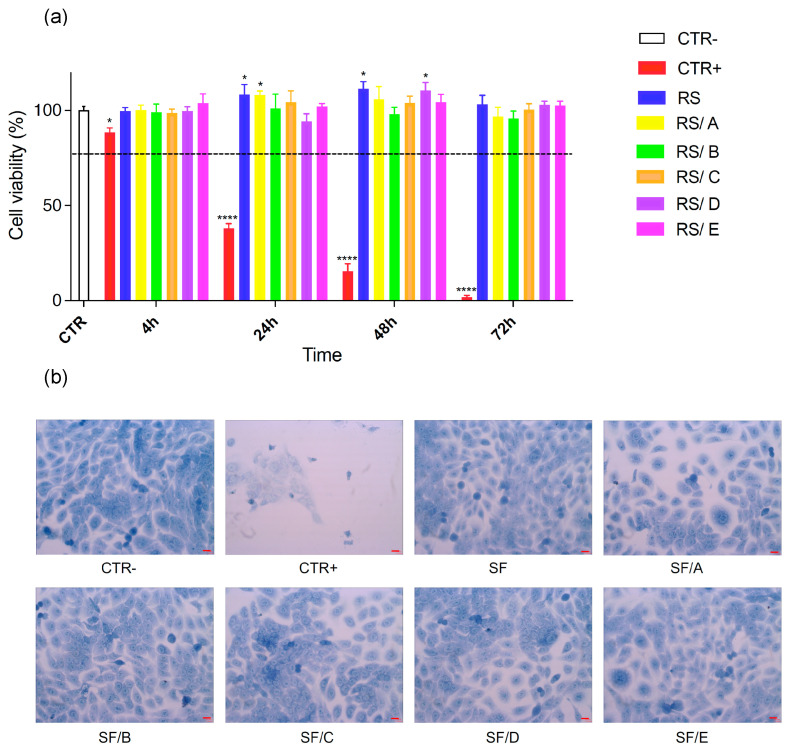
(**a**) MTT assay on HaCaT cells after 4, 24, 48, and 72 h of 220 s irradiation. The SF and SF/DNA labels indicate the viability of the HaCaT cells covered with these films during the irradiation. In the statistical analysis, the significance thresholds are * *p* < 0.05 and **** *p* < 0.0001. (**b**) Optical microscopy images of HaCaT cells after 24 h of 220 s irradiation with the protection of SF and SF/DNA films. Negative and positive controls were also reported. Red lines in each picture indicate 1.00 µm.

HaCaT cells were exposed to combinations of spectral bands from 230 nm to 780 nm for 220 s. The 6.95 kJ/m^2^ UVB exposure corresponds to 1 h and 28 min in mid-summer, according to Lauder, New Zealand, as a reference measurement station (45° S, 170° E, altitude 370 m) [48]. The exposure to the solar spectrum resulted in a significant time-dependent decrease in metabolic activity of the cells detectable 24 h post-irradiation (Figure 4a) according to the results previously reported by Plitta-Michalak et al. with exposure to single spectral bands [33], whereas we did not observe a significant cytotoxic effect after 4 h of irradiation with 88.4% of cell viability (data reported in red in Figure 4a). Irradiation of HaCaT cells with the protection of SF and SF/DNA films did not cause the same toxic profile as for the unprotected ones, demonstrating that these films modulated the cell metabolic activity. In particular, we found that the coatings increased the metabolic activity with respect to the negative control (data reported in white in Figure 4a) at different post-irradiation times; this result is not due to a higher number of cells. Ghasemi et al. [49] demonstrated that cell density may change the level of produced formazan in every single cell, increasing the cell enzymatic activity. Accordingly, our findings could be explained considering that both SF and SF/DNA patches shield the cells from UVC and UVB radiations while the other components of the solar spectrum are able to penetrate, stimulating important physiological activities in the skin. Further studies in this regard are under investigation to better understand which biochemical pathway is implicated. In general, within the statistical significance, the neat SF also exhibited a significant protective effect both after 24 and 48 h. The HaCaT cell morphology was also investigated by using methylene blue staining (Figure 4b). In detail, non-irradiated cells (CTR- sample) are organized in a uniform layer made of polygonal and flattened cells; the cells appear intensely stained with well-outlined borders. This is proof that cells are alive and active; cell–cell interactions via integrins have been deeply demonstrated to modulate epidermal morphogenesis and integrity [50]. On the contrary, irradiated cells (CTR+) were numerically less (more than 50% died) and weakly stained. Moreover, after 220 s of irradiation, it was evident that cells lost their own morphology, being smaller than CTR- and exhibiting a changed shape, which became crumpled and round with cytoplasmic retraction and membrane characteristic of apoptotic/necrotic cells. Cells covered with SF and SF/DNA films were mostly flattened and spread with protrusions. In general, protected cells are similar to those of CTR-but slightly less stained. These findings proved the UV-blocking activity of both neat SF and SF/DNA, suppressing biochemical UV-induced cell damage.

## 4. Conclusions

In this study, we present a simple solution processing of DNA salmon sperm and silk fibroin to produce adhesive patches with thermal stability properties. The results suggest that the DNA embedded in the SF matrix retains its conformation. Moreover, the synergistic combination of SF and DNA acts as a UV-blocking filter preserving the viability of human keratinocyte HaCaT cells under exposure to the UV component of the solar spectrum. Because of the facile processability in the solution, we envision the utilization of such bio-materials for the realization of environmentally sustainable sunscreens, which might pave the way for the formulation of various hybrid materials that exploit the photothermal properties of 2D nanostructures to prevent bacterial infections. These findings may expand wound healing management.

## Data Availability

Data can be made available upon reasonable request from the corresponding author.

## Supplementary Materials

The following supporting information can be downloaded at: https://www.mdpi.com/article/10.3390/jfb14050280/s1, Video S1. The flexibility of SF/DNA sample after soaking in PBS. Video S2. Adhesiveness characterization of the SF/A sample. Figure S1. Transmittance variation of SF/DNA samples after the subtraction of the spectrum of SF in the infrared region. Figure S2. Optical microscopy images of SF and SF/D samples. Figure S3. MTT assay on HaCaT cells after 24 h of 440 s irradiation time.
