# Supplementary material for: Development of Salmon Sperm DNA/Regenerated Silk Bio-Based Films for Biomedical Studies on Human Keratinocyte HaCaT Cells under Solar Spectrum
Source: J Funct Biomater. 2023 May 18;14(5):280. doi: 10.3390/jfb14050280 (PMC10219309; doi:10.3390/jfb14050280)
Supplement: Supplementary file 1 [file jfb-14-00280-s001.zip › Figures S1-S3.pdf]

# SUPPLEMENTARY MATERIALS

# Development of Salmon Sperm DNA/Regenerated Silk Bio-Based Films for Biomedical Studies on Human Keratinocyte HaCaT Cells under Solar Spectrum

**Maria Rachele Ceccarini [1], Francesca Ripanti [2], Veronica Raggi [3], Alessandro Paciaroni [2], Caterina Petrillo [2], Lucia Comez [4], Kevin Donato [5,6], Matteo Bertelli [5,6], Tommaso Beccari [1] and Luca Valentini [7,*]**

1. Department of Pharmaceutical Sciences, University of Perugia, 06123 Perugia, Italy
2. Department of Physics and Geology, University of Perugia, Via Alessandro Pascoli, 06123 Perugia, Italy
3. Polo Scientifico Didattico, University of Perugia, Strada di Pentima 4, 05100 Terni, Italy
4. Istituto Officina dei Materiali-IOM, National Research Council-CNR, Via Alessandro Pascoli, 06123 Perugia, Italy
5. MAGI EUREGIO SCS, Via Maso della Pieve, 60/A, 39100 Bolzano, Italy
6. MAGISNAT, Atlanta Tech Park, 107 Technology Parkway, Peachtree Corners, GA 30092, USA
7. Civil and Environmental Engineering Department, University of Perugia, Strada di Pentima 6, 05100 Terni, Italy

* Correspondence: luca.valentini@unipg.it

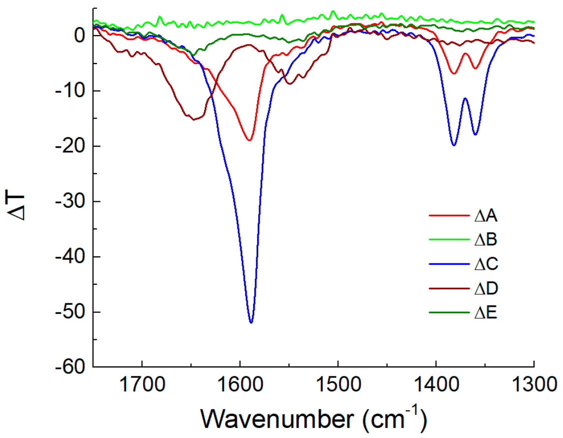


Figure S1. Transmittance variation of SF/DNA samples after the subtraction of the spectrum of SF in the infrared region.

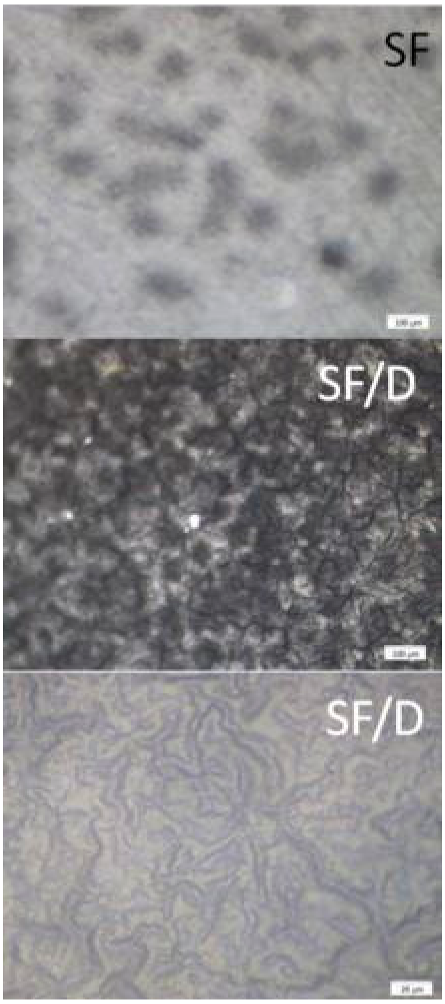


Figure S2. Optical microscopy images of SF and SF/D samples.

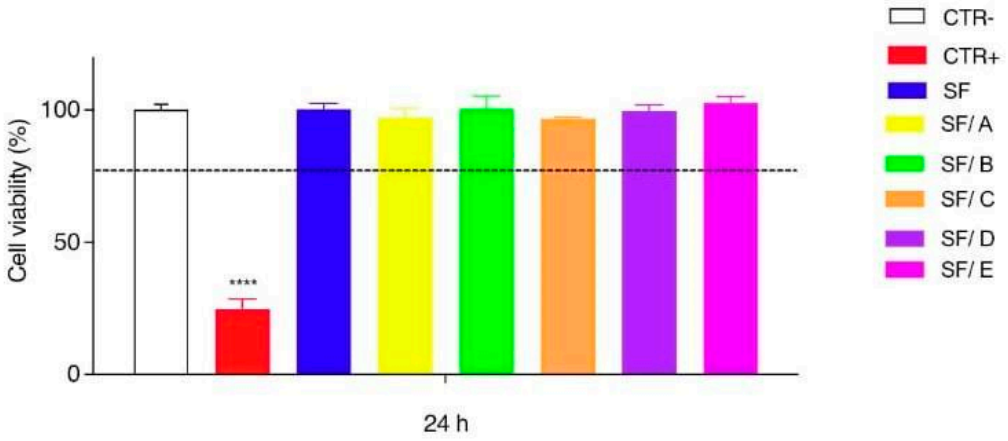


Figure S3. MTT assay on HaCaT cells after 24 h of 440 s irradiation. The SF and SF/DNA labels indicate the viability of the HaCaT cells covered with these films during the irradiation.
